# Genome-wide association mapping revealed a diverse genetic basis of seed dormancy across subpopulations in rice (*Oryza sativa* L.)

**DOI:** 10.1186/s12863-016-0340-2

**Published:** 2016-01-25

**Authors:** Risper Auma Magwa, Hu Zhao, Yongzhong Xing

**Affiliations:** National Key Laboratory of Crop Genetic Improvement and National Center of Plant, Gene Research (Wuhan), Huazhong Agricultural University, Wuhan, 430070 China; Hubei Collaborative Innovation Center for Grain Industry, Hubei, China

**Keywords:** Seed dormancy, Germination percentage, After-ripening, Association mapping, Haplotype analysis

## Abstract

**Background:**

Seed dormancy is an adaptive trait employed by flowering plants to avoid harsh environmental conditions for the continuity of their next generations. In cereal crops, moderate seed dormancy could help prevent pre-harvest sprouting and improve grain yield and quality. We performed a genome wide association study (GWAS) for dormancy, based on seed germination percentage (GP) in freshly harvested seeds (FHS) and after-ripened seeds (ARS) in 350 worldwide accessions that were characterized with strong population structure of indica, japonica and Aus subpopulations.

**Results:**

The germination tests revealed that Aus and indica rice had stronger seed dormancy than japonica rice in FHS. Association analysis revealed 16 loci significantly associated with GP in FHS and 38 in ARS. Three out of the 38 loci detected in ARS were also detected in FHS and 13 of the ARS loci were detected near previously mapped dormancy QTL. In FHS, three of the association loci were located within 100 kb around previously cloned GA/IAA inactivation genes such as *GA2ox3*, *EUI1* and *GH3*-*2* and one near dormancy gene, *Sdr4*. In ARS, an association signal was detected near ABA signaling gene *ABI5*. No association peaks were commonly detected among the sub-populations in FHS and only one association peak was detected in both indica and japonica populations in ARS. *Sdr4* and *GA2OX3* haplotype analysis showed that Aus and indica II (IndII) varieties had stronger dormancy alleles whereas indica I (IndI) and japonica had weak or non-dormancy alleles.

**Conclusion:**

The association study and haplotype analysis together, indicate an involvement of independent genes and alleles contributing towards regulation and natural variation of seed dormancy among the rice sub-populations.

**Electronic supplementary material:**

The online version of this article (doi:10.1186/s12863-016-0340-2) contains supplementary material, which is available to authorized users.

## Background

Seed dormancy, a phenomenon in which mature and viable seeds fail to germinate under conditions favorable for its germination in a specified period of time, is a very complicated trait controlled by both environmental as well as genetic factors arising from both maternal and embryonic tissues [1–3]. In nature, seed dormancy is an adaptive trait that is used by the wild species to delay germination until the environmental factors favorable for the survival of their offspring is available [2]. In a controlled environment, seed dormancy is measured based on germination percentages, rates or index as the percentage of the number of seeds germinated out of the total numbers of seeds planted in a specified number of days (usually seven to fourteen days for germination percentage) [4, 5]. Dormancy is one of the traits among the cereal crops that have undergone domestication and could be a desirable trait as it can help prevent pre-harvest sprouting hence improved grain yield and quality [6, 7]. Since deep dormancy prevents germination and weak dormancy exposes the seeds to pre-harvest sprouting, moderate dormancy levels would be desirable in order to avoid the extremes of the dormancy levels [8].

In cultivated rice species, mean dormancy periods varies from one cultivar to another [9]. The depth of dormancy is affected by the seed maturity stage [10, 11] and the environmental factors such as the temperature during seed ripening [12], the day length [13], the storage temperature [14, 15] and seed moisture content during the dry after-ripening period [15] among others. Besides the environmental factors, seed dormancy is also regulated by a number of plant hormones such as abscisic acid, gibberellic acid, auxin, ethylene and brassinosteroids [16, 17].

Studies conducted in Arabidopsis revealed key seed maturation regulators including *FUS3*, *LEC1 and LEC2*, DAG1 *and ABI3* [18–20]. Molecular studies on mutations in *HISTONE MONOUBIQUITINATION* (*HUB1*) identified a decreased dormancy in Arabidopsis seeds due to transcriptional control via effects on chromatin structure [21]. In Arabidopsis *DELAY OF GERMINATION 1* (*DOG1*) QTL was cloned and was involved in embryonic dormancy [22]. KYP/SUVH4, a mediator of H3 lysine 9 dimethylation was demonstrated as a negative regulator of seed dormancy in Arabidopsis [23]. *RDO5* was found to positively regulate seed dormancy by suppressing transcript levels of *APUM9* and *APUM11* in Arabidopsis [24]. Chromatin remodeling was shown to correlate positively with *DOG1* expression in response to dormancy cycling in the soil seed bank in Arabidopsis [25]. In rice, *qSD7*–*1*, a clustered QTL (*qSD7–1/qPC7*) was delimited to the pleiotropic Rc locus and found to control seed dormancy by regulating ABA biosynthetic pathway in rice [26]. *Sdr4*, a global regulator of seed maturation was cloned in rice and was positively regulated by OsVP1 [27].

A number of seed dormancy QTL has been reported in cultivated rice and wild rice [28–33]. Gramene QTL database for rice has documented 165 dormancy QTL including *qDOR*, *qSD* and *sdr* loci (http://www.gramene.org). The QTL mapped in the 12 chromosomes of rice except chromosome 10 included cluster QTL such as *qSD7*/*qPC7*, qSD1–2/qPH1 and qSD7–2/qPH7 [34, 35]. The successful use of QTL linkage mapping promoted the studies of the genetic architecture of various traits in rice; however it had a major limitation due to its restriction of allelic diversity between bi-parents leading to low resolution [36, 37].

GWAS tends to solve the shortcomings of QTL linkage mapping since it does not require the development of a specific segregating population to detect QTL. A larger number of gene pools and millions of genome-wide SNPs from next generation sequencing used in GWAS can narrow down confidence intervals for the loci detected with higher genomic resolutions [38]. GWAS successes have not been without limitations such as the genetic architecture of the trait being controlled either by rare variants with large effects on the phenotype or common variants with small phenotypic effect [39, 40]. The likelihood of false positive associations due to strong or complete linkage of rare variants with other non-causative rare variants further reduces GWAS successes [41, 42]. A large and geographically diverse sample size or a large sample of local population with higher phenotypic diversity hence maximized genetic variation or minimized genetic heterogeneity within the sample, respectively provides a solution to GWAS shortcomings [43]. Combining several SNPs in a region into a single indicator variable as a composite genotype can reduce the detection of rare variants [44]. The use of mixed models have also minimized the detection of false positive associations by accounting for the resultant phenotypic covariance that is due to genetic relatedness [45, 46]. The success of GWAS in detecting genes of agronomic importance such as grain quality, grain yield, morphology, stress tolerance, and nutritional quality in rice, have demonstrated its usefulness in identifying more genome-wide genes contributing to seed dormancy in rice [47–50]. In Arabidopsis, an integration of GWAS and transcriptomic analysis identified HD2B as a negative regulator of seed dormancy during cold induced dormancy cycling [51].

In the present study, we used the GWAS strategy in a global collection of 350 rice accessions to evaluate the seed dormancy variations based on seed GP within and among the Aus, indica and japonica subpopulations. Our results identified 16 and 38 significant loci associated with seed dormancy in freshly harvested seeds and after-ripened seeds respectively. The detection of previously identified dormancy gene (*Sdr4*), *qSD7–1*, *ABI5*, GA/IAA catabolic genes and previously mapped QTL near the association loci in our study validated the reliability of our association mapping. This study also revealed the influence of different alleles in controlling dormancy among various cultivated rice groups. The detected association loci could be mined and used to improve pre-harvest sprouting tolerance by marker assisted selection (MAS) approach.

## Results

### Phenotypic evaluations and heritability

**Fig. 1:**
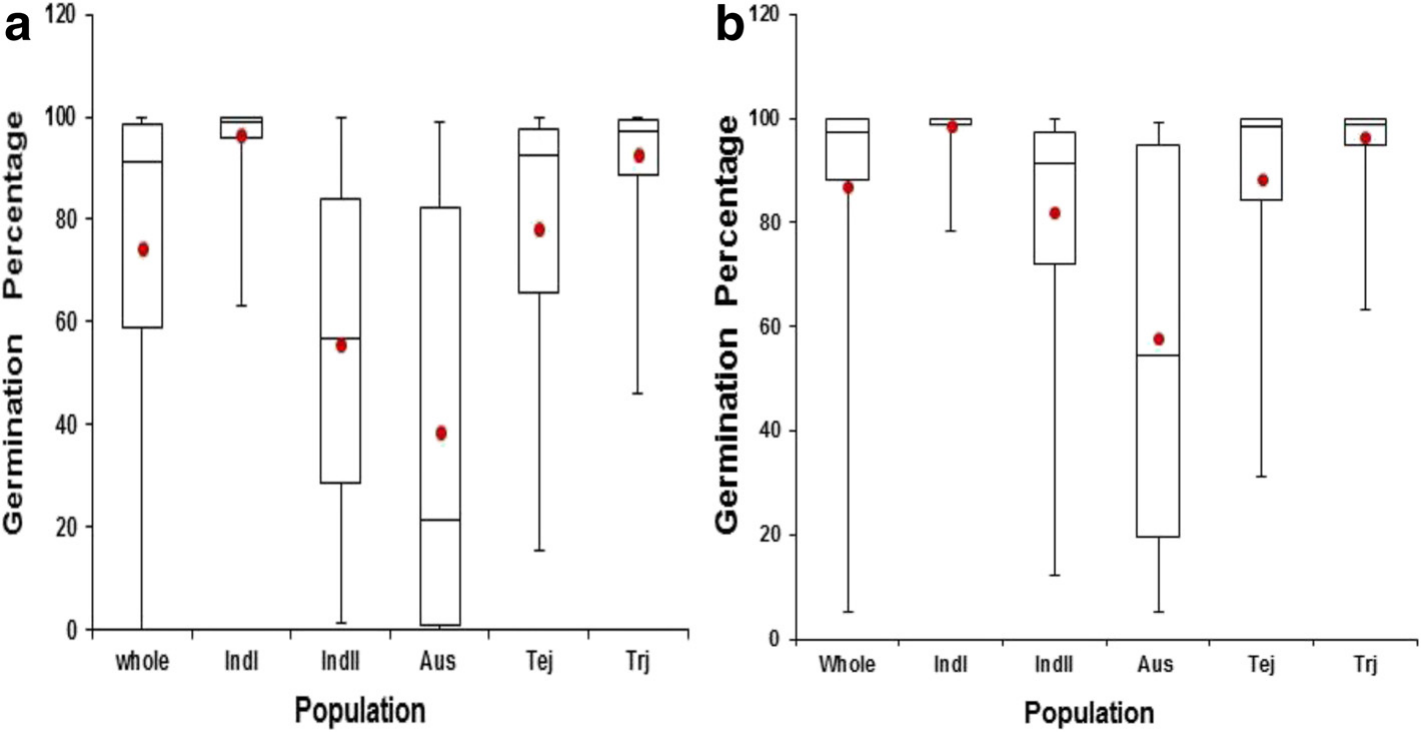
Phenotype distribution of GP represented in boxplots. The upper part of the box represents the 3^rd^ quantile and the lower part the 1^st^ quantile with the line in between as the Median (2^nd^ quantile). The whiskers represent the highest and the lowest data points. The means for GP are shown in red dots. **a** Boxplot showing distribution of GP in freshly harvested seeds; **b** Boxplot showing distribution of GP in After-ripened seeds

A collection of 350 accessions of *O. sativa* collected from various parts of the world was used in this study. The germplasm consisted of indica, japonica, Aus subpopulations and intermediates (Additional file 1). The indica population was further sub-divided into indica I (IndI) and indica II (IndII) subpopulations and japonica into temperate japonica (Tej) and tropical japonica (Trj) subpopulations. In this diversity panel, FHS of Aus varieties had the lowest mean GP (38.6 %). The greatest range of GP was observed in Aus and IndII varieties (Table 1). The mean GP difference was largest between IndI (96.7 %) and IndII which had 55.5 % (Fig. 1a). No such significant difference was observed between Tej and Trj which had mean GPs of 78.1 % and 92.6 % respectively. These results signified that some genotypes could be characterized with strong seed dormancy.

**Table 1:** Germination percentages of the freshly harvested seeds and after- ripened seeds in the whole population and subpopulations of indica, japonica and Aus

Seeds	Terms	Whole	IndI	IndII	Tej	Trj	Aus
FHS	Range	0–100	63.3–100.0	1.3–100.0	15.2–100	45.5–100	0–98.8
FHS	M ± SD	74.5 ± 32.0	96.7 ± 6.2	55.5 ± 31.5	78.1 ± 25.9	92.6 ± 10.8	38.6 ± 40.1
ARS	Range	5.2–100	78.4–100	12.1–100	31.2–100	63.1–100	5.2–100
ARS	M ± SD	87.3 ± 22.9	98.6 ± 3.7	82.2 ± 21.1	88.2 ± 18.2	96.5 ± 6.8	57.9 ± 37.0

*FHS* freshly harvested seeds, *ARS* after-ripened seeds, *SD* standard deviation, *M* mean germination percentage, *IndI* indica I, *IndII* indica II, *Tej* temperate japonica, *Trj* tropical japonica

For the ARS, Aus varieties had the lowest mean GP (57.9 %) with IndI having the highest (98.6 %). IndII, Tej and Trj had mean GP of 82.2, 88. 2 and 96.5 % respectively (Table 1). On average, the mean GP of each subpopulation were significantly increased in ARS as compared to their corresponding mean GP in the FHS with exception of IndI and Trj (Fig. 1b). In addition, the variation in GP among the five subpopulations in ARS was much lower (57.9 % - 98.6 %) as compared to that of FHS (38.6 % - 96.7 %) (Table 1). Obviously, seed dormancy had been released to some extent or completely broken during the two-month after-ripening period depending on the variety (Additional files 2 and 3). Furthermore, the heritability (*H*^*2*^) of GP was 92.0 % and above in any of the populations.

### Association mapping in FHS

**Fig. 2:**
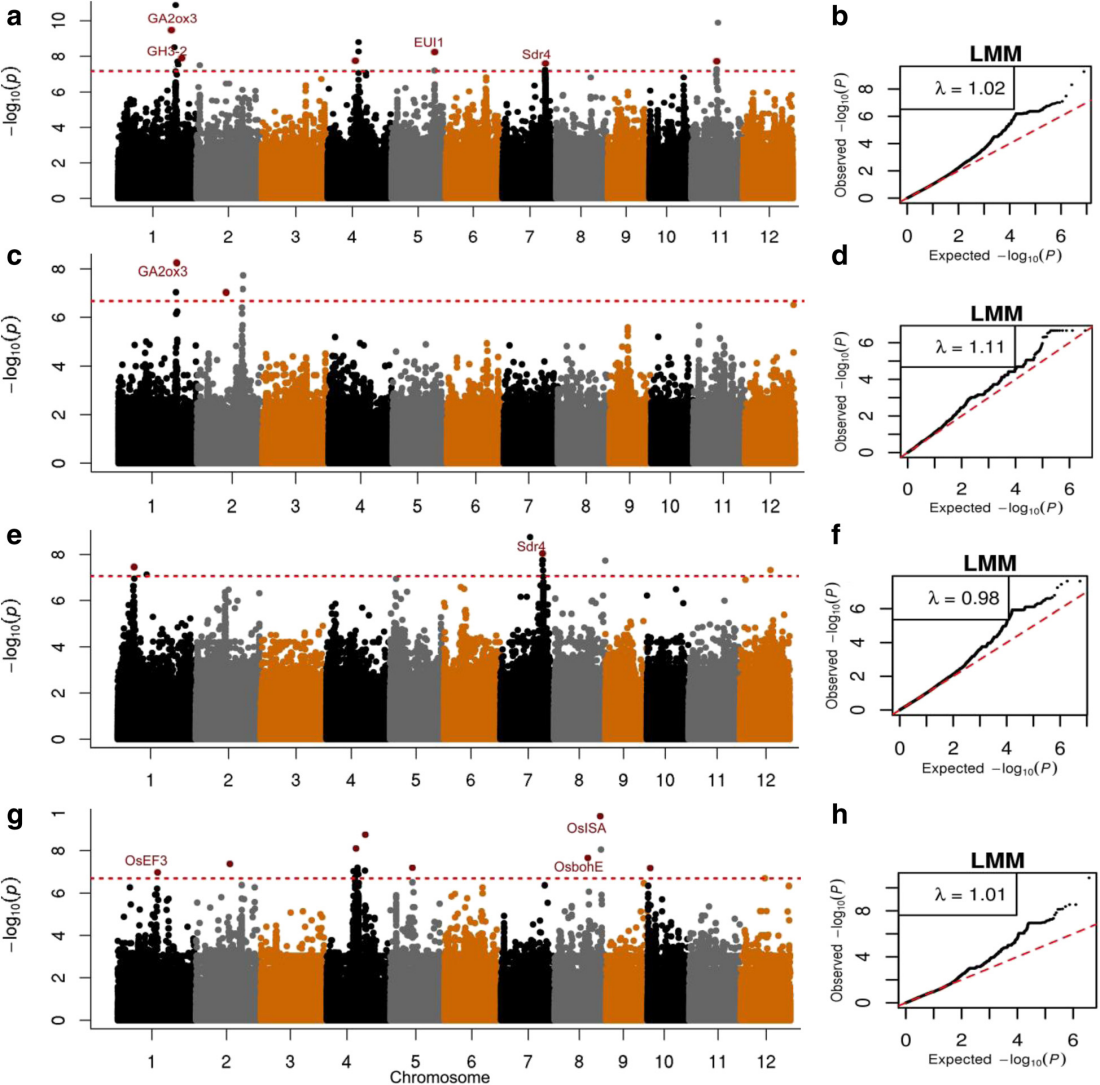
Genome wide association mapping of GP in the freshly-harvested seeds of rice populations using LMM method. Identified significant loci are shown in red dots. Known genes identified within 100 kb near association peaks are indicated in red. The Manhattan plots for GP shows –log_10_ *P*-values from genome-wide scan plotted against the position on each of the 12 chromosomes. The dashed line indicates the genome-wide significance thresholds, *P* = 6.6 × 10^−8^ (whole population), 2.1 × 10^−7^ (Aus), 8.7 × 10^−8^ (indica) and 2.0 × 10^−7^ (japonica). The horizontal axis in quantile-quantile plots shows –log_10_ transformed expected-values and the vertical axis indicates –log_10_ transformed observed *P*-values. **(a**) and (**b**) are Manhattan plot and quantile-quantile plot for GP, respectively in whole population. **(c) ** and (**d**) are Manhattan plot and quantile-quantile plot for GP respectively, in Aus. (**e) ** and (**f**) are Manhattan plot and quantile-quantile plot for GP respectively, in indica. **g** and (**h**) are Manhattan plot and quantile-quantile plot for GP respectively, in japonica

**Fig. 3:**
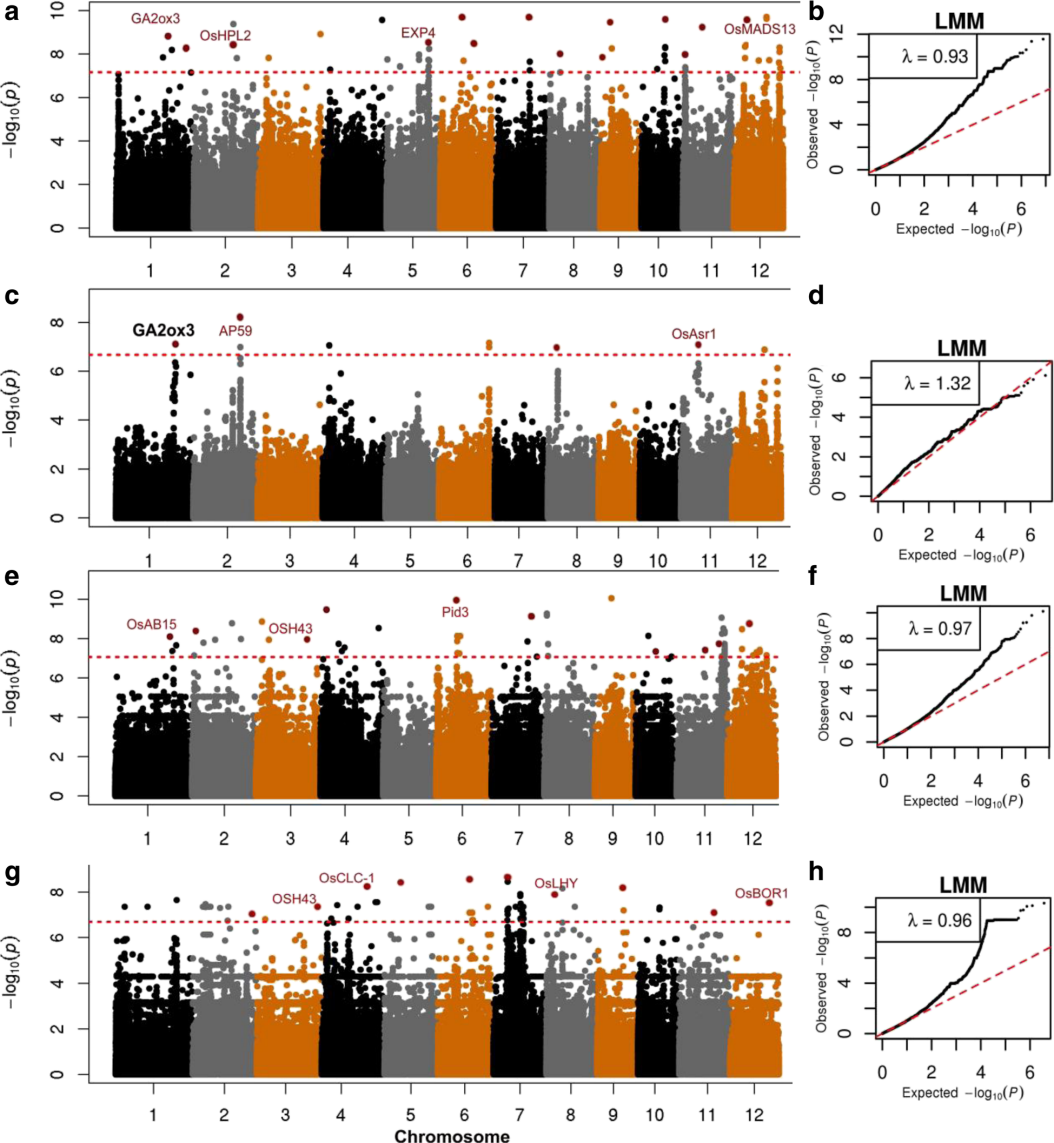
Genome wide association mapping of GP in the after-ripened seeds of various rice populations using LMM method. Identified significant loci are shown in red dots. Known genes identified within 100Kb near association peaks are indicated in red. The Manhattan plots for GP shows –log_10_ *P*-values from genome-wide scan plotted against the position on each of the 12 chromosomes. The dashed line indicates the genome-wide significance thresholds, *P* = 6.6 × 10^−8^ (whole population), 2.1 × 10^−7^ (Aus), 8.7 × 10^−8^ (indica) and 2.0 × 10^−7^ (japonica). The horizontal axis in quantile-quantile plots shows –log_10_ transformed expected-values and the vertical axis indicates –log_10_ transformed observed *P*-values (**a**) and (**b**) are Manhattan plot and quantile-quantile plot for GP respectively, in whole population. **(c)** and (**d**) are Manhattan plot and quantile-quantile plot for GP respectively, in Aus. (**e) ** and (**f**) are Manhattan plot and quantile-quantile plot for GP respectively, in indica. (**g) ** and (**h**) are Manhattan plot and quantile-quantile plot for GP respectively, in japonica

To determine QTL associated with seed dormancy, we carried out GWAS on GP in FHS of indica, japonica and Aus subpopulations independently and in the whole panel using linear mixed model (LMM). The Manhattan plots and quantile-quantile plots for the GP in FHS and ARS using LMM are shown in Figs. 2 and 3. Considering that linkage disequilibrium (LD) decay in cultivated rice was extended from 100 kb to 200 kb [47, 52, 53] the association peaks falling within a region of less than 150 kb were considered as one association peak. In consequence, a total of 16 association signals were identified for GP in FHS (Table 2).

**Table 2:** Genome-wide significant association signals of GP in the freshly harvested seeds using LMM method

Pop	Signals	Lead SNP^b^	Chr	Position	*P* -Value	Contr %	Known gene	Dis (kb)	QTL	Reference
Whole	FHS1.1^ca^	sf0131727984	1	31727984	5.3E-10	1.6	GA2ox3	66		
	FHS1.2	sf0132299347	1	32299347	1.8E-08	18.9	GH3–2	−75		
	FHS4.1	sf0415712552	4	15712552	2.7E-08	6.7				
	FHS5.1	sf0523711156	5	23711156	4.9E-09	8.7	EUI1	−35	qDGR5b	[33]
	FHS7.0^c^	sf0723792996	7	23792996	5.1E-08	18.3	Sdr4	3		
	FHS11^a^	sf1114334640	11	14334640	3.3E-08	1.4				
Aus	FHS1.1^ca^	sf0131727984	1	31727984	8.2E-09	6.0	GA2ox3	66		
	FHS2.1	sf0217781998	2	17781998	1.9E-07	71.1				
Indica	FHS1.3	sf0116084564	1	16084564	7.4E-08	0.3				
	FHS7.0^c^	sf0723792996	7	23792996	1.2E-08	44.9	Sdr4	3		
Japonica	FHS1.4	sf0121684641	1	21684641	1.1E-07	1.7	OsEF3	−43		
	FHS2.2	sf0217958009	2	17958009	6.8E-08	5.0				
	FHS4.2	sf0416131386	4	16131386	7.2E-09	10.7				
	FHS4.3	sf0421133500	4	21133500	2.5E-09	4.7				
	FHS5.2^a^	sf0510583389	5	10583389	8.4E-08	3.8				
	FHS8.1	sf0822226957	8	22226957	2.2E-08	36.8	OsbohE	−9		
	FHS8.2	sf0825914157	8	25914157	1E-10	4.1	OsISA	−16		
	FHS10	sf1008134438	10	8134438	6.7E-08	2.5				

FHS signals for germination percentage in freshly harvested seeds, followed by the chromosome and the signal number in the chromosome. Lead SNPs ID, the first two digits after sf indicate the chromosome number followed by the position on chromosome. Contr., contribution to the phenotype variance. Dis, the distance from known gene locus to the lead SNP with the negative sign representing upstream

^a^commonly detected signals in FHS and ARS

^b^for more information see Additional file 6

^c^Commonly detected signals between subpopulations

Six signals (*FHS1.1, FHS1.2, FHS4.1, FHS5.1, FHS7and FHS11*) were detected for GP in the whole population on chromosomes 1, 4, 5, 7 and 11 (Table 2). They individually explained 1.4–18.9 % of the GP variance. There were 2, 2 and 8 lead SNPs associated with GP in the subpopulations Aus, indica and japonica, respectively (Table 2). The association loci (*FHS2.1*) detected in Aus explained the highest GP variance of 71.1 %. Two associations (*FHS1.3* and *FHS7*) were detected in indica rice. *FHS7* was identified in both indica and whole population whereas *FHS1.1* in Aus and whole population. None of the eight signals detected in japonica subpopulation were detected in the whole population or in any other subpopulation. The associations explained more of GP variance within the subpopulations than in whole population. For example, *FHS7* explained 18.3 % of GP variance in the whole population, whereas it explained 44.9 % in indica subpopulation.

### Association mapping in ARS

Accordingly, we conducted GWAS on GP in ARS and a total of 38 associations were identified. Fourteen signals were detected in the whole population across the 12 chromosomes except chromosomes 3 and 4 (Table 3). They individually explained 0.1–29.3 % of GP variance. Four, 10 and 10 signals were detected in Aus, indica and japonica subpopulations respectively. The signal *ARS1.1* was detected in both whole and Aus populations while *ARS3* was detected in both indica and japonica. Even though the three signals *ARS1.1* (whole and Aus), *ARS11*.2 (whole) and *ARS5.2* (japonica) were detected in both FHS and ARS, their phenotype contribution was lower in ARS than in FHS, for example *ARS1.1* in Aus contributed to 6 % GP variance in FHS and only 0.7 % in ARS (Table 3). The signal in Aus (*ARS8.2)* contributed to the highest GP variance (40.1 %). Thirteen out of the 38 signals were harbored within the regions of previously mapped dormancy QTL which probably could be the candidates for these associations.

**Table 3:** Genome-wide significant association signals of GP in the after-ripened seeds using LMM method

Pop	Signals	Lead SNP^b^	Chr	Position	*P* value	Contr%	Known gene	Dis (kb)	QTL	Reference
Whole	ARS1.1^ca^	sf0131727984	1	31727984	1E-09	0.3	GA2ox3	66		
	ARS1.2	sf0138588391	1	38588391	6E-09	29.3			qSD1	[79]
	ARS2.1	sf0206686399	2	6686399	4.1E-09	0.1	OsHPL2	−56	qSD2	[32]
	ARS5.1	sf0523494718	5	23494718	1.5E-09	9.1	EXP4	−60		
	ARS6.1	sf0611491573	6	11491573	1E-10	0.5			qSD6	[79]
	ARS6.2	sf0620628649	6	20628649	1.3E-09	2.4				
	ARS7.1	sf0720587413	7	20587413	1E-10	1.8				
	ARS8.1	sf0807777822	8	7777822	2.6E-08	0.7				
	ARS9.1	sf0905593232	9	5593232	7.3E-10	1.6				
	ARS9.2	sf0914236447	9	14236447	3.7E-08	3.9				
	ARS10.1	sf1019156688	10	19156688	2.4E-10	0.6				
	ARS11.1	sf1101407923	11	1407923	2.5E-08	0.1			qDOR11–1	[80]
	ARS11.2^a^	sf1114328194	11	14328194	5.6E-10	1.2				
	ARS12.1	sf1207745486	12	7745486	1.1E-10	2.9	OsMADS13	52		
Aus	ARS1.1^ca^	sf0131706848	1	31706848	8.4E-08	0.7	GA2ox3	87		
	ARS2.2	sf0226365962	2	26365962	6.2E-09	27.3	AP59	50		
	ARS8.2	sf0804981132	8	4981132	2.6E-07	40.1				
	ARS11.3	sf1103208304	11	3208304	1.7E-07	9.5	OsAsr1	66	qDOR11–1	[80]
Indica	ARS1.3	sf0137198693	1	37198693	9.8E-09	1.7	OsABI5	23	qSD.1	[31]
	ARS2.3	sf0201212724	2	1212724	8.6E-09	2.0				
	ARS3^c^	sf0332024688	3	32024688	1.7E-08	9.3	OSH43	−61	qDOR3–3	[80]
	ARS4.1	sf0403783716	4	3783716	1.6E-10	30.2				
	ARS6.3	sf0613057982	6	13057982	9.8E-11	7.0	Pid3	−1		
	ARS7.2	sf0726194419	7	26194419	4.6E-09	8.4				
	ARS10.2	sf1018639995	10	18639995	7.2E-08	0.9				
	ARS11.4	sf1119110821	11	19110821	5.7E-08	8.0			qDOR11–3	[80]
	ARS11.5	sf1126973978	11	26973978	1.5E-08	1.1			qDOR11–6	[80]
	ARS12.2	sf1214076931	12	14076931	5.7E-10	5.4				
Japonica	ARS2.4	sf0232512303	2	32512303	2E-07	6.6				
	ARS3	sf0331989391	3	31989391	5.2E-08	0.7	OSH43	−26	qDOR3–3	[80]
	ARS4.2	sf0421860784	4	21860784	3.9E-09	36.6	OsCLC-1	25		
	ARS5.2^a^	sf0510583389	5	10583389	3.5E-09	2.1				
	ARS6.4	sf0619595095	6	19595095	3.1E-09	4.2				
	ARS7.3	sf0708015120	7	8015120	2.3E-09	4.3			qSD7–1	[79]
	ARS8.3	sf0803353408	8	3353408	1.3E-08	8.3	OsLHY	11		
	ARS9.3	sf0922932869	9	22932869	6.1E-09	2.5				
	ARS11.6	sf1122384837	11	22384837	2E-07	8.7			qDOR11–4	[80]
	ARS12.3	sf1223239793	12	23239793	6E-08	0.5	OsBOR1	−20	qSD12	[79]

*ARS* signals for germination percentage in after-ripened seeds, followed by the chromosome and the signal number in the chromosome, *Lead SNPs ID* the first two digits after sf indicate the chromosome number followed by the position on chromosome, *Contr* contribution to the phenotype variance, *Dis* the distance from known gene locus to the lead SNP with negative sign representing upstream

^a^commonly detected signals in FHS and ARS

^b^for more information see Additional file 7

^c^Commonly detected signals between subpopulations

### Genes and QTL around the putative peak positions

The phytohormones ABA and GA have been implicated to significantly control seed dormancy by the intrinsic balance of their biosynthesis and catabolism respectively [54]. Thus, a higher ratio of ABA to GA leads to dormancy and vice versa [55]. We searched for the dormancy related genes including ABA, GA and other plant hormones regulating dormancy such as auxin around the association peaks. Since the LD decay in cultivated rice was extended from 100 kb to 200 kb [47, 52, 53], the genes for dormancy related hormones within the 100-kb regions upstream and downstream of the association peaks in this study were considered to be the possible candidate genes for seed dormancy. Around the 16 association peaks detected in FHS, two GA related genes, one auxin related gene and one dormancy related gene were identified (Table 2). *FHS7* (sf0723792996) was located 3 kb upstream of the first cloned seed dormancy gene in rice, *Sdr4* [27] in both indica and whole populations. *GA2ox3*, a GA catabolic gene [56] was located in the position of 66 kb downstream of *FHS1.2* and was identified in both Aus and whole population. *EUI1,* a GA inactivation gene [57, 58] was located 35 kb upstream of *FHS5* in the whole population. *GH3–2*, an IAA (major form of auxin in rice) inactivating gene that acts to catalyze the formation of an IAA amino acid conjugate leading to the suppression of expansin gene [59], was detected 75 kb upstream of *FHS1.1* in the whole population (Table 2). In ARS, the ABA related gene *ABI5* was detected 23 kb downstream of *ARS1.3* and was found to be involved in ABA signaling and in the regulation of LEA genes during seed maturation and germination [60]. *OsHPL2* was detected near *ARS2.1* and plays a role in inhibition of seed germination [61, 62]. OsAsr1, believed to be involved in ABA signaling in response to osmotic stress [63] was detected 66 kb downstream of *ARS11.3* (Table 3).

In addition to known cloned genes, one and eleven previously mapped QTL were detected in the regions harboring the association loci in FHS and ARS respectively. The signal *FSGP5* in FHS was harbored in the regions of *qDGR5b* on chromosome 1 (Table 2). The thirteen signals flanked within the regions of previously mapped QTL in ARS were spread across the chromosomes 1, 2, 3, 6,7,11 and 12 (Table 3).

### *Sdr4* haplotypes analysis

In order to ascertain the contributions of *Sdr4* towards seed dormancy, we analyzed its haplotypes within the coding region. There were 4 SNPs within the coding region of *Sdr4*, 1 synonymous and 3 non-synonymous (Additional file 4a). The 3 non-synonymous SNPs resulted into 3 haplotypes (Hap1- Hap3) among the 350 accessions. Hap1 was the dominant haplotype present in 70, 61.4 and 100 % of Aus, indica and japonica varieties respectively. Hap2 was present in 30 % Aus and 30 % indica varieties. Hap3 was uniquely identified in indica at 8.6 % (Table 4). Comparison analysis within indica subpopulation revealed significant differences between Hap1 and Hap2 and between Hap1 and Hap3. There was no significant difference between Hap2 and Hap3 in indica. Significant difference was also observed between Hap1 and Hap2 in Aus (Table 4). Varieties possessing Hap2 in Aus and indica subpopulations had the lowest mean GP compared to Hap1 counterparts that had the highest mean GP. No variation of *Sdr4* was observed in japonica rice (Table 4).

**Table 4:** Haplotype diversity within *Sdr4* and *GA2OX3* genes and their mean GP in subpopulations

Gene	Hap	Indica		Aus		Japonica	
		No	GP %	No	GP %	No	GP %
Sdr4	Hap1	121	92.2 ± 15.9B	21	50.4 ± 38.2B	95	85.4 ± 20.9
	Hap2	59	52.2 ± 30.5A	9	11.0 ± 30.9A		
	Hap3	17	57.4 ± 27.9A				
GA2OX3	Hap1	167	73.0 ± 30.6B	20	21.5 ± 34.0A	11	83.6 ± 14.3A
	Hap2	26	98.1 ± 3.5A	10	72.8 ± 28.0B		
	Hap3	4	92.7 ± 8.0A			83	85.5 ± 21.7A

*Hap* Haplotype, *SD* standard deviation, *No* number of cultivars within a given population, *GP* germination percentage, means followed by different letters are significantly different at P˂ 0.05

### *GA2OX3* haplotypes analysis

*GA2ox3* is a GA catabolism gene that catalyzes the oxidation of GA_20_ to GA2_9_ and GA_29_ to GA_29_-catabolites [56]. It is responsible for the homeostatic regulation of biologically active GA concentration in rice; hence its expression leads to reduced GA levels and suppressed germination or growth. Due to its direct involvement in the GA pathway and its subsequent detection near association peaks in Aus and whole population, we conducted SNPs search within its genomic DNA and found a total of 22 SNPs including 3 non-synonymous SNPs among the 350 accessions. The 3 non-synonymous SNPs namely sf0131794745, sf0131794598 and sf0131795793 resulted into amino acid changes from Leucine to Valine, Valine to isoleucine and Alanine to Valine, respectively (Additional file 4a). The haplotype analysis using the non-synonymous SNPs resulted into a total of 3 haplotypes (Hap1 to Hap3). Here we compared the difference in GP among the 3 major haplotypes (Table 4). Hap1 was commonly found in 66.7, 85.6, and 11.7 % of Aus, indica and japonica (only Trj) varieties respectively. Hap2 was found in 33.3 % of Aus and 13.3 % indica (IndI) and was absent in japonica. 88.3 % of japonica varieties had Hap3 (Additional file 4b). Comparison analysis within Aus subpopulation revealed a significant difference in GP between Hap1 and Hap2. In indica there was a significant difference between Hap1 and Hap2 while the difference between Hap2 and Hap3 was not significant. There was no significant difference between Hap1 and Hap3 of japonica. Except for the Hap1 of Aus varieties, which had the lowest mean GP of 21.5 %, all other haplotypes showed a higher mean GP of above 70 % across the various subpopulations (Table 4) indicating that Hap1 allele could probably be functioning only in Aus and not in other subpopulations.

## Discussion

### Diverse genetic basis of seed dormancy in indica, japonica and Aus subpopulations

Seed dormancy is a complex trait controlled by genetic and environmental conditions during seed development and storage [2, 9]. Thus temperature during grain filling in rice is an important determinant of levels of seed dormancy. The harvest time in relation to stage of ripening as well as the levels of temperature and humidity during storage is equally important in dormancy maintenance and release. Thus in order to minimize environmental effects experiment-wise, only 350 accessions whose seed development stages experienced similar temperature and humidity conditions in the field were kept for testing seed dormancy. In addition, the panicles for each accession that emerged within 2–3 days were uniformly harvested 32 days after heading, which minimized the environmental noise within accessions.

Our results indicated a lower GP of FHS in Aus and IndII at about 39 and 55 % respectively, whereas IndI and tropical japonica had very high GPs of more than 90 %. The temperate japonica subpopulation had a GP of about 78 %. On average, most Aus accessions and a number of IndII varieties had strong seed dormancy compared to IndI subpopulation, which had no seed dormancy and the japonica subpopulation which had weak dormancy. Whereas seed dormancy was diverse within indica subpopulation, no big difference in GP was observed within japonica subpopulation. It is believed that Tej and Trj have a close genetic relationship with a lower genetic diversity [64] and that Tej was derived from Trj [65, 66]. Thus the minor differences in GP between the two japonica subpopulations could probably be as a result of the low genetic diversity. A previous study showed that Aus have a smaller geographic distribution and a very high genetic diversity coupled with adaptive traits [67]. Therefore, the lowest GP levels and higher phenotypic contribution (up to 71 %) in Aus were probably due to the diverse genetic differentiation. In addition, there were 16 associations detected in FHS which were unique to their specific subpopulations. More signals were expected to be detected in indica and Aus than in japonica due to lower GP levels and wider GP variance experienced in indica and Aus compared to japonica. However the results were the reverse. This case could be explained by few major QTL like *Sdr4* identified in indica and Aus subpopulations and several minor QTL in japonica*.*

Previous findings have shown that 4–6 weeks could readily release dormancy in rice seeds stored at 20–30 °C at 11 % moisture content [15, 43]. In ARS there was a sharp GP increase in IndII and Aus with indica and japonica subpopulations having a mean GP of above 80 % in ARS while Aus had an increased GP of 59.9 % up from 38.6 %, an indication that the two months of after-ripening was able to completely break the dormancy or significantly release seed dormancy of many accessions in our study. There were 38 signals detected in ARS out of which 10, 4 and 10 were detected in indica, Aus and japonica respectively. Of these signals, only one signal *(ARS3)* was commonly detected in both indica and japonica. These results together indicated that different genes/alleles controlled seed dormancy in various rice sub-groups probably due to their divergent evolution and domestication processes.

### Early and late detectable signals controlling seed dormancy

Dormancy QTL have been categorized into three based on the detection of their main effect throughout the after-ripening period [30, 68]. The QTL included those with constant effect which were detectable in FHS and stayed throughout the after-ripening duration, early detectable effects which influenced germination of FHS and became less effective after a few weeks of after-ripening and late detectable QTL whose effect on germination were detectable at a later time during the after-ripening period. In this categorization of the QTL, the genetic interactions and the dormancy allele background had to be considered [30]. Our GWAS study identified a total of 16 and 38 association peaks in FHS and ARS respectively. Only three signals (*FHS1.1, FHS11* and *FHS5.2)* out of the 16 signals in FHS could be detected in ARS while the remaining 13 associations disappeared. One signal in FHS and 13 signals in ARS were detected within the regions of previously mapped QTL indicating that these previously mapped QTL harboring the association signals could probably be the candidates for these associations.

It was also interesting to note that 35 out of the 38 signals detected in ARS were not detected in FHS posing a question “why were there more signals detected in ARS than in FHS when seed dormancy was released to a larger extent?” Probably the dormancy QTL categorization provides an answer to this question. The three commonly detected signals in FHS and ARS probably kept functioning in freshly harvested seeds through to the after-ripening seeds but their genetic effect was decreased with time like in *Sdr4*. The FHS signals lost in ARS could probably be related to early detectable dormancy effects and or weak dormancy alleles that influenced the germination of FHS and became less effective after the two months after-ripening. The 35 association signals newly detected in ARS including the 13 signals harbored in the regions of previously mapped QTL were probably related to the late effect detectable QTL. Transcriptomic study in *A. thaliana* revealed a separate genetic mechanism underlying dormancy establishment and after-ripening (AR) in seeds, and that AR genes were down-regulated in freshly harvested seeds and up-regulated in stored seeds [69]. Thus, we may conclude that there exist independent genes controlling seed dormancy in FHS and ARS.

### Candidate genes for seed dormancy

Dormancy in seeds has been studied in relation to failure of seeds to germinate in a specified period of time and by examining the expression levels of ABA, GA and other growth related phytohormones in the wild type and mutants [3, 18, 19]. Hundreds of seed dormancy QTL have been detected by linkage mapping (http://www.gramene.org) but only dormancy genes, *Sdr4* [27], *qSD7-1* [26] and the endosperm imposed seed dormancy QTL, qSD1–2 [70] have been molecularly cloned in rice. Our association mapping resulted into 16 lead SNPs in FHS seeds; two of which were located less than 100 kb near GA inactivation genes (*GA2ox3 and EUI1*), 1 near auxin inactivation gene (*GH3–2*) and one near *Sdr4*. The GA genes were reported to regulate rice growth and panicle architecture by regulating the concentration of biologically active GA [56–58, 71]. The auxin related gene (*GH3–2*) was reported to inactivate IAA by catalyzing the formation of an IAA amino acid conjugate resulting in the suppression of expansins [59]. It is most likely that these genes have effect on seed dormancy and could be the candidates for these associations. Although the signal *ARS1.2* was detected more than 200 kb away from *qSD1–2*/*GA20ox-2*, we propose *GA20ox-2* to be the possible candidate of *ARS1.2.* Loss-of-function mutations of the *OsGA20ox2* resulted into reduced GA levels, which slowed down tissue morphogenesis, delayed ABA accumulation and subsequent maturation programs hence decreased dormancy at harvest [70]. The failure to detect the genes directly involved in ABA pathway near the associated loci in FHS but instead a few ABA related genes like *ABI5, OsAsr1* and *OsHPL2* (implicated in ABA signaling pathway) in ARS, could be an indication that dormancy maintenance and release in rice is independent of ABA levels though it plays a significant role in these mechanisms. In Arabidopsis it was demonstrated that ABA is not a dormancy-specific factor in imbibed rice seeds but rather a growth regulator of seed dormancy and germination [72]. It was of interest to notice that *GA2ox3* was detected near association loci in both FHS and ARS in Aus, an indication that *GA2ox3* plays a crucial role in dormancy maintenance and is a stable gene. In barley, after-ripening was found to promote expression of *HvGA2ox3* in imbibed after-ripened seeds [73]. In Sorghum a transcriptional study in an imbibed dormant seed harvested 30 days after pollination (DAP) revealed an early activity of GA synthesis that was suppressed by increased deactivation rate by *SbGA2ox3* and *SbGA2ox1* which were highly expressed. The expression of these two catabolic genes however, disappeared in imbibed dormant seed harvested 42 DAP [74]. Thus a further follow-up and closer study of GA catabolic genes in relation to their direct involvement in seed dormancy maintenance should be conducted using direct mutagenesis by genome editing technique or transcriptomic technique since in the past, more studies have been directed towards ABA and to some extent GA synthesis genes leaving aside the GA catabolic genes which could be of equal importance as ABA in regards to seed dormancy.

### *Sdr4* and *GA2ox3* haplotypes for breeding pre-harvest sprouting resistance variety

The haplotype analysis within *Sdr4* and *GA2ox3* genes revealed that different alleles controlled seed dormancy among different rice populations. For example, *Sdr4 Hap1* conferred low dormancy; *Hap3* unique only to indica had moderate dormancy whereas *Hap2* conferred strong dormancy especially in Aus. All japonica varieties possessed *Hap1* alleles. These results supported the previous study on *Sdr4* that there are three different alleles *Sdr4-n*, *Sdr4-k* and *Sdr4-k*’ and all japonica varieties carrying *Sdr4-n* alleles conferred low dormancy, whereas *Sdr4-n* and *Sdr4-k* were widely distributed in indica [27]. Even though *Hap2* conferred strong dormancy, there were some accessions in *Hap2* that had higher GP and some accessions in *Hap1* that had lower GP. This occurrence was concluded to be as a result of the genetic interaction between *Sdr4* and a modifier gene, *OsVP1* [27]. We noted that all IndI varieties with exception of four varieties had *Hap1* alleles while only 24 % of IndII had *Hap1* alleles (Additional file 4b), an indication that IndI was extremely selected for reduced dormancy during domestication, eventually rendering them non-dormant. Accordingly, the genetic interactions between the *GA2ox3* haplotypes can be researched further in order to understand the relation between the *GA2ox3* haplotypes in conferring seed dormancy. Thus, upon its validation*, GA2ox3* can be considered for breeding pre-harvest sprouting resistant rice varieties since *GA2ox3* was only detected near association peaks in Aus, which notably had the lowest GP as compared to any other subpopulations and also its inhibiting effect on germination persisted throughout the after-ripening period.

## Conclusions

In conclusion, this study revealed that different genes/alleles conferred dormancy in the various subpopulations of rice in FHS and ARS. The association loci may provide a rich source of information about the natural genetic variations underlying the evolution, domestication and breeding of indica, Aus and japonica rice in relation to seed dormancy and other adaptive traits. The major association signals could be useful in improving the non-dormant IndI and Trj varieties to possess moderate seed dormancy by crossing them with strong dormant varieties from Aus and IndII using marker assisted selection (MAS) breeding approach.

## Methods

### Plant materials

A worldwide rice collection consisting of 529 rice accessions [75] were grown in the experimental station of Huazhong Agricultural University, Wuhan in May 2014 for seed dormancy evaluation. Seven plants were planted in each row with spacing of 16.5 cm between plants within a row and 26.4 cm between rows. Field management was conducted according to the standard agronomic practices. The five middle plants in each row were tagged for heading dates, harvested and used for examining seed dormancy. In order to minimize the noise on environmental effect, 350 accessions whose seed development was completed in high temperature and high humidity conditions were selected for seed dormancy evaluation.

### Phenotype assessment for seed dormancy

The accessions were grown to maturity in the field and the heading dates of two early heading panicles from each of the five middle plants were individually recorded, and the panicles were tagged every day. The tagged panicles of each accession that headed in the same dates (2–3 day heading date interval) were harvested 32 days after heading, pooled together and used to score the germination percentages. The variation of heading date was large from June 15^th^ to August 30^th^ across the whole population. In order to minimize the environmental noise, only the accessions that flowered between 1^st^ July 2014 and 5^th^ August 2014 were used for germination analysis. The average daily temperature during this period ranged from 23 to 28 °C with an average humidity of between 70–95 %.

The panicles that flowered on the same date and/or having one to two day heading date intervals were harvested from the five middle plants of each accession and dried at 30 °C for 24 h; the seeds removed from panicles and pooled together and separated into two batches. The first batch of seeds for each accession was surface sterilized with 0.6 % sodium hypochlorite solution for 15 min, rinsed five times with distilled water and pre-germinated by soaking in distilled water with changing of water every day for 48 h at 30 °C. 100 imbibed seeds from each accession were transferred into 9 cm Petri-plates lined with wet filter paper in three replicates and placed in a growth chamber set at 28 °C for 14 h light and 22 °C for 10 h dark with 100 % relative humidity for 7 days. The seed was considered germinated when the radicle or coleoptile reached a length of ≥2 mm. GP was scored as the percentage of the number of seeds germinated in the total numbers of seeds in the plate at the first 7 days. The seeds from the second batch were stored at room temperature (~25 °C) for two months to break dormancy by way of after-ripening, after which the seeds were used for germination tests as described above. The germination percentage results were presented as the mean of the germination percentages obtained from the three replicates of 100 seeds ± standard deviation (SD).

### Association mapping

Next generation sequencing for the accessions collection was conducted in the previous study [76], and population structure was modeled as a random effect in linear mixed model (LMM) using the kinship (K) matrix and GWAS was performed using LMM provided by the FaST-LMM programme [77]. The numbers of SNPs used for GWAS for the whole population and each subpopulation were as follows: whole population 3,916,415, Aus 1,925,362, indica 2,767,159 and japonica 1,857,845 while considering only the SNPs with minor allele frequency of ≥0.05 and the varieties with the minor allele frequency of ≥6 in a population. However, Some SNPs were completely linked, thereby causing redundancy in GWAS. Thus the number of informative SNPs (*M*) was used to calculate the effective number of independent SNPs (*Me*) after a modified Bonferroni correction [78]. The effective numbers of independent SNPs (Additional file 5) were then used in calculating the genome-wide significance thresholds for GWAS based on a nominal value of 0.05 for LMM resulting into a stringent genome-wide significant threshold value of 6.6 × 10^−8^, 8.7 × 10^−8^, 2.0 × 10^−7^ and 2.0 × 10^−7^ in the whole population, subpopulations indica, japonica and Aus respectively.

### *Sdr4* and *GA2ox3* Haplotype analysis

The whole genomic DNA analysis of *Sdr4* and *GA2ox3* genes among the 350 accessions resulted into 4 and 22 SNPs respectively (http://ricevarmap.ncpgr.cn). Only the non-synonymous SNPs within the coding regions of these genes were used for haplotype analysis.

### Availability of supporting data

The data sets supporting the results of this article are included within the article and its additional files. The original data sets used in this study are available upon request as part of the data is not for public.

## Additional files

Additional file 1:
**Neighbor-joining tree of the 350 rice accessions with reference to GP: A neighbor-joining tree showing the divergent groups of the 350 rice accessions used in this study with reference to germination percentage (GP).** (PDF 123 kb) Additional file 2:
**List of twenty most dormant accessions that retained their dormancy in After-ripened seeds.** This table contains names of twenty most dormant accessions, sub- population, country of origin and the germination percentages in FHS and ARS. (PDF 95 kb) Additional file 3:
**List of most dormant accessions that lost their dormancy in After-ripened seeds.** This table contains names of most dormant accessions, sub- population, country of origin and the germination percentages in FHS and ARS. (PDF 105 kb) Additional file 4:
**Non-synonymous SNPs in Sdr4 and GA2ox3 genes in 350 accessions used in our study.** This file contains two tables (a) and (b). Table (a) contains the non-synonymous SNPs in Sdr4 and GA2ox3 genes, the SNPs position within the chromosomes, the minor and major alleles for the SNPs and the Amino acid changes in relation to the nucleotide change from major to minor alleles. The tables also shows the haplotype diversity within these two genes. Table (b) shows the number of accessions in individual sub-populations possessing any of the Sdr4 and GA2ox3 haplotypes. (PDF 101 kb) Additional file 5:
**Estimated effective number of SNPs and significant Thresholds in populations: This table shows the effective number of independent SNPs** (***Me***) **after a modified Bonferroni correction calculated using informative SNPs** (***M***) **in Whole population, Aus, indica and japonica populations.** (PDF 85 kb) Additional file 6:
**The Genome-wide association mapping results for Germination Percentage (GP) of the freshly harvested seeds (FHS) in whole, Aus, indica and japonica populations.** The figure shows neighbor-joining tree, histogram of the phenotypes (GP), quantile-quantile plot of the expected null distribution and the observed P-value and the Manhattan plots of GP of freshly harvested seeds in populations using LMM and LR methods. (PDF 795 kb) Additional file 7:
**The Genome-wide association mapping results for Germination Percentage (GP) of the after-ripened seeds (ARS) in whole, Aus, indica and japonica populations.** The figure shows neighbor-joining tree, histogram of the phenotypes (GP), quantile-quantile plot of the expected null distribution and the observed P-value and the Manhattan plots of GP of after-ripened seeds in populations using LMM and LR methods. (PDF 823 kb)

## References

[1] Bewly JD (1997). Seed germination and dormancy. Plant Cell.

[2] Li B, Foley ME (1997). Genetic and molecular control of Seed Dormancy. Trends Plant Sci.

[3] Baskin JM, Baskin CC (2004). Classification system for seed dormancy. Seed Sci Res.

[4] Baskin CC, Baskin JM (1998). Seeds: ecology, biogeography, and evolution of dormancy and germination.

[5] Veasey EA, Karasawa MGM, Santos PP, Rosa MS, Mamanie E, Oliveira GCX (2004). Variation in the loss of seed dormancy during after-ripening of wild and cultivated Rice Species. Ann Bot.

[6] Harlan JR, de Wet JMJ, Price EG (1973). Comparative evolution of cereals. Evolution.

[7] Gubler F, Millar AA, Jacobsen JV (2005). Dormancy release, ABA and pre-harvest sprouting. Curr Opin Plant Biol.

[8] Bewley JD, Black M (1994). Seeds- physiology of development and germination.

[9] Roberts EH (1961). Dormancy of rice seed. I. The distribution of dormancy periods. J Exp Bot.

[10] Roberts EH (1962). Dormancy in rice seed. III. The influence of temperature, moisture and gaseous environment. J Exp Bot.

[11] Anderson JA, Sorrells ME, Tanksley SD (1993). RFLP analysis of genomic regions associated with resistance to pre-harvest sprouting in wheat. Crop Sci.

[12] Ikehashi H (1972). Induction and test of dormancy of rice seeds by temperature condition during maturation. Japan J Breed.

[13] Takahashi N (1997). Inheritance of seed germination and dormancy. Science of rice plant: genetics.

[14] Roberts EH (1965). Dormancy in rice seed. IV. Varietal responses to storage and germination temperatures. J Exp Bot.

[15] Cohn MA, Hughes JA (1981). Seed dormancy in red rice (Oryza sativa). I. Effect of temperature on dry-afterripening. Weed Sci.

[16] Koornneef M, Bentsink L, Hilhorst H (2002). Seed dormancy and germination. Curr Opin Plant Biol.

[17] Finkelstein RR, Davies PJ (2004). The role of hormones during seed development and Germination. Plant Hormones – biosynthesis, signal transduction, action!.

[18] Rohde A, Kurup S, Holdsworth M (2000). ABI3 emerges from seed. Trends Plant Sci.

[19] Monke G, Altschmied L, Tewes A, Reidt W, Mock HP, Baumlein H (2004). Seed-specific transcription factors *ABI3* and *FUS3:* molecular interaction with DNA. Planta.

[20] Gualberti G, Papi M, Bellucci L, Ricci I, Bouchez D, Camilleri C (2002). Mutations in the Dof zinc finger genes *DAG2* and *DAG1* influence with opposite effects germination of Arabidopsis seeds. Plant Cell.

[21] Liu Y, Koornneef M, Soppe WJ (2007). The absence of histone H2B monoubiquitination in the Arabidopsis *hub1* (rdo4) mutant reveals a role for chromatin remodeling in seed dormancy. Plant Cell.

[22] Bentsink L, Jowett J, Hanhart CJ, Koornneef M (2006). Cloning of DOG1, a quantitative trait locus controlling seed dormancy in Arabidopsis. Proc Natl Acad Sci U S A.

[23] Zheng J, Chen FY, Wang Z, Cao H, Li X, Deng X (2012). A novel role for histone methyltransferase KYP⁄SUVH4 in the control of Arabidopsis primary seed dormancy. New Phytol.

[24] Xiang Y, Nakabayashi K, Ding J, He F, Bentsink L, Soppe WJJ (2014). REDUCED DORMANCY5 Encodes a Protein Phosphatase 2C that Is Required for Seed Dormancy in Arabidopsis. Plant Cell.

[25] Footitt S, Müller K, Kermode AR, Finch-Savage WE (2015). Seed dormancy cycling in Arabidopsis: chromatin remodeling and regulation of DOG1 in response to seasonal environmental signals. Plant J.

[26] Gu XY, Foley ME, Horvath DP, Anderson JV, Feng J, Zhang L (2011). Association between seed dormancy and pericarp color is controlled by a pleiotropic gene that regulates abscisic acid and flavonoid synthesis in weedy Red rice. Genet.

[27] Sugimoto K, Takeuchi Y, Ebana K, Miyao A, Hirochika H, Hara N (2010). Molecular cloning of *Sdr4*, a regulator involved in seed dormancy and domestication of rice. Proc Natl Acad Sci U S A.

[28] Lin SY, Sasaki T, Yano M (1998). Mapping quantitative trait loci controlling seed dormancy and heading date in rice, *Oryza sativa* L., using backcross inbred lines. Theor Appl Genet.

[29] Dong Y, Tsuzuki E, Kamiunten H, Terao H, Lin D, Matsuo M (2003). Identification of quantitative trait loci associated with pre-harvest sprouting resistance in rice (*Oryza sati*va L.). Field crops Res.

[30] Gu XY, Kianian SF, Foley ME (2004). Multiple loci and epistases control genetic variation for seed dormancy in weedy rice (*Oryza sativa*). Genet.

[31] Wan JM, Cao YJ, Wang CM, Ikehashi H (2005). Quantitative trait loci associated with seed dormancy in rice. Crop Sci.

[32] Jiang L, Cao YJ, Wang CM, Zhai HQ, Wan JM, Yoshimura A (2003). Detection and analysis of QTL for seed dormancy in rice (*Oryza sativa L*.) using RIL and CSSL population. Acta Genet Sin.

[33] Li W, Xu L, Bai X, Xing Y (2010). Quantitative trait loci for seed dormancy in rice. Euphytica.

[34] Gu XY, Turnipseed EB, Foley ME (2008). The qSD12 locus controls offspring tissue-imposed seed dormancy in rice. Genet.

[35] Ye H, Beighley DH, Feng J, Gu XY (2013). Genetic and physiological characterization of two clusters of quantitative trait loci associated with seed dormancy and plant height in rice. G3 (Bethesda).

[36] Borevitz JO, Nordborg M (2003). The impact of genomics on the study of natural variation in Arabidopsis. Plant Physiol.

[37] Korte A, Farlow A (2013). The advantages and limitations of trait analysis with GWAS: a review. Plant Methods.

[38] Hindorff LA, Sethupathy P, Junkins HA, Ramos EM, Mehta JP, Collins FS (2009). Potential etiologic and functional implications of genome-wide association loci for human diseases and traits. Proc Natl Acad Sci U S A.

[39] Asimit J, Zeggini E (2010). Rare variant association analysis methods for complex traits. Annu Rev Genet.

[40] Gibson G (2011). Rare and common variants: twenty arguments. Nat Rev Genet.

[41] Dickson SP, Wang K, Krantz I, Hakonarson H, Goldstein DB (2010). Rare variants create synthetic genome-wide associations. PLoS Biol.

[42] Wray NR, Purcell SM, Visscher PM (2011). Synthetic associations created by rare variants do not explain most GWAS results. PLoS Biol.

[43] Li Y, Huang Y, Bergelson J, Nordborg M, Borevitz JO (2010). Association mapping of local climate sensitive quantitative trait loci in Arabidopsis thaliana. Proc Natl Acad Sci U S A.

[44] Feng T, Zhu X (2012). Detecting rare variants. Methods Mol Biol.

[45] Yu J, Pressoir G, Briggs WH, Vroh BI, Yamasaki M, Doebley JF (2006). A unified mixed-model method for association mapping that accounts for multiple levels of relatedness. Nat Genet.

[46] Listgarten J, Lippert C, Kadie CM, Davidson RI, Eskin E, Heckerman D (2012). Improved linear mixed models for genome-wide association studies. Nat Methods.

[47] Huang X, Wei X, Sang T, Zhao Q, Feng Q, Zhao Y (2010). Genome-wide association studies of 14 agronomic traits in rice landraces. Nat Genet.

[48] Zhao K, Tung CW, Eizenga GC, Wright MH, Ali ML, Price AH (2011). Genome-wide association mapping reveals a rich genetic architecture of complex traits in *oryza sativa*. Nat Commun.

[49] Norton GJ, Douglas A, Lahner B, Yakubova E, Guerinot ML, Pinson SRM (2014). Genome wide association mapping of grain arsenic, copper, molybdenum and zinc in rice (Oryza sativa L.) grown at four international field sites. PLoS One.

[50] Eizenga GC, Ali ML, Bryant RJ, Yeater KM, McClung AM, McCouch SR (2014). Registration of the ‘Rice Diversity Panel 1’ for genome-wide association studies. J Plant Registrations.

[51] Yano R, Takebayashi Y, Nambara E, Kamiya Y, Seo M (2013). Combining association mapping and transcriptomics identify HD2B histone deacetylase as a genetic factor associated with seed dormancy in Arabidopsis thaliana. Plant J.

[52] Mather KA, Caicedo AL, Polato NR, Olsen KM, McCouch S, Purugganan MD (2007). The extent of linkage disequilibrium in rice (Oryza sativa L.). Genet.

[53] McNally KL, Childs KL, Bohnert R, Davidson RM, Zhao K, Ulat VJ (2009). Genome-wide SNP variation reveals relationships among landraces and modern varieties of rice. Proc Natl Acad Sci U S A.

[54] Ali-Rachedi S, Bouinot D, Wagner MH, Bonnet M, Sotta B, Grappin P (2004). Changes in endogenous abscisic acid levels during dormancy release and maintenance of mature seeds: studies with the Cape Verde Islands ecotype, the dormant model of *Arabidopsis thaliana*. Planta.

[55] Cadman CS, Toorop PE, Hilhorst HW, Finch-Savage WE (2006). Gene expression profiles of *Arabidopsis* Cvi seeds during dormancy cycling indicate a common underlying dormancy control mechanism. Plant J.

[56] Sakai M, Sakamoto T, Saito T, Matsuoka M, Tanaka H, Kobayashi M (2003). Expression of novel rice gibberellin 2-oxidase gene is under homeostatic regulation by biologically active gibberellins. J Plant Res.

[57] Zhu Y, Nomura T, Xu Y, Zhang Y, Peng Y, Mao B (2006). Elongated uppermost internode encodes a cytochrome P450 monooxygenase that epoxidizes gibberellins in a novel deactivation reaction in rice. Plant Cell.

[58] Luo A, Qian Q, Yin HF, Liu XQ, Yin CX, Lan Y (2006). EUI1, encoding a putative cytochrome P450 monooxygenase, regulates internode elongation by modulating gibberellin response in rice. Plant Cell Physiol.

[59] Fu J, Liu H, Li Y, Yu H, Li X, Xiao J (2011). Manipulating broad-spectrum disease resistance by suppressing pathogen-induced auxin accumulation in rice. Plant Physiol.

[60] Finkelstein R, Lynch T (2000). The Arabidopsis abscisic acid response gene *ABI5* encodes a basic leucine zipper transcription factor. Plant Cell.

[61] Gardner HW, Dornbos DLJ, Desjardins A (1990). Hexanal, trans-2-hexenal, and trans-2-nonenal inhibit soybean, *Glycine max*, seed germination. J Agric Food Chem.

[62] Chehab EW, Raman G, Walley JW, Perea JV, Banu G, Theg S (2006). Rice HYDROPEROXIDE LYASES with unique expression patterns generate distinct aldehyde signatures in Arabidopsis. Plant Physiol.

[63] Vaidyanathan R, Kuruvilla S, Thomas G (1998). Characterization and expression pattern of an abscisic acid and osmotic stress responsive gene from rice. Plant Sci.

[64] Ni J, Colowit P, Mackill D (2002). Evaluation of genetic diversity in rice subspecies using microsatellite markers. Crop Sci.

[65] Glaszmann JC (1987). Isozymes and classification of Asian rice varieties. Theor Appl Genet.

[66] Zhang Q, Maroof M, Lu T, Shen B (1992). Genetic diversity and differentiation of *Indica* and *Japonica* rice detected by RFLP analysis. Theor Appl Genet.

[67] Garris AJ, Tai TH, Coburn J, Kresovich S, McCouch S (2005). Genetic structure and diversity in Oryza sativa L. Genet.

[68] Alonso-Blanco C, Bentsink L, Hanhart CJ, Vries HBE, Koornneef M (2003). Analysis of natural allelic variation at seed dormancy loci of Arabidopsis thaliana. Genet.

[69] Carrera E, Holman T, Medhurst A, Dietrich D, Footitt S, Theodoulou FL (2008). Seed after-ripening is a discrete developmental pathway associated with specific gene networks in Arabidopsis. Plant J.

[70] Ye H, Feng JH, Zhang LH, Zhang JF, Mispan MS, Cao ZQ (2015). Map-based cloning of seed dormancy1–2 identified a gibberellin synthesis gene regulating the development of endosperm-imposed dormancy in rice. Plant Physiol.

[71] Miura K, Ikeda M, Matsubara A, Song XJ, Ito M, Asano K (2010). OsSPL14 promotes panicle branching and higher productivity in rice. Nat Genet.

[72] Gianinetti A, Vernier P (2007). On the role of abscisic acid in seed dormancy of red rice. J Exp Bot.

[73] Gubler F, Hughes T, Waterhouse P, Jacobsen J (2008). Regulation of dormancy in barley by blue light and after-ripening: effects on abscisic acid and gibberellin metabolism. Plant Physiol.

[74] Rodriguez MV, Mendiondo GM, Cantoro R, Auge GA, Luna V, Masciarelli O (2012). Expression of seed dormancy in grain sorghum lines with contrasting pre-harvest sprouting behavior involves differential regulation of gibberellin metabolism genes. Plant Cell Physiol.

[75] Chen W, Gao Y, Xie W, Gong L, Lu K, Wang W (2014). Genome-wide association analyses provide genetic and biochemical insights into natural variation in rice metabolism. Nat Genet.

[76] Zhao H, Yao W, Ouyang Y, Yang W, Gong W, Wang GW, et al. RiceVarMap: a comprehensive database of rice genomic variations. Nucleic Acids Res. 2014. 43 doi: 10.1093/nar/gku894. 10.1093/nar/gku894 PMC4384008 25274737

[77] Lippert C, Listgarten J, Liu Y, Kadie CM, Davidson RI, Heckerman D (2011). FaST linear mixed models for genome-wide association studies. Nat Methods.

[78] Li MX, Yeung JM, Cherny SS, Sham PC (2012). Evaluating the effective numbers of independent tests and significant *p*-value thresholds in commercial genotyping arrays and public imputation reference datasets. Hum Genet.

[79] Gu XY, Kianian SF, Foley ME (2005). Phenotypic selection for seed dormancy introduced a set of adaptive haplotypes from weedy into cultivated rice. Genet.

[80] Cai HW, Morishima H (2000). Genomic regions affecting seed shattering and seed dormancy in rice. Theor Appl Genet.

