# Supplementary material for: Genome-wide association mapping revealed a diverse genetic basis of seed dormancy across subpopulations in rice (Oryza sativa L.)
Source: BMC Genet. 2016 Jan 25;17:28. doi: 10.1186/s12863-016-0340-2 (PMC4727300; doi:10.1186/s12863-016-0340-2)
Supplement: Additional file 1: — Neighbor-joining tree of the 350 rice accessions with reference to GP: A neighbor-joining tree showing the divergent groups of the 350 rice accessions used in this study with reference to germination percentage (GP). (PDF 123 kb) [file 12863_2016_340_MOESM1_ESM.pdf]

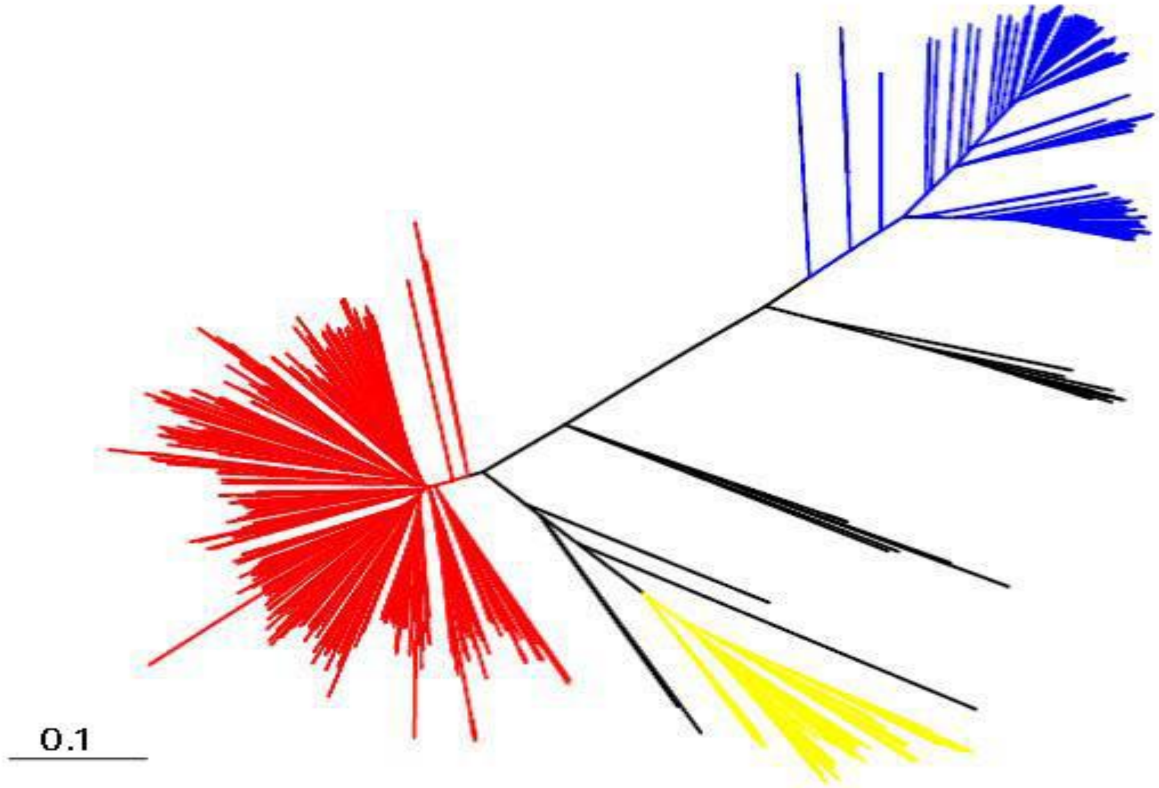

Additional file 5. Neighbor-joining tree of 350 rice accessions with reference to GP. The four subgroups identified include Indica (red), Japonica (blue), Aus (yellow) and intermediate (black). The scale bar indicates the simple matching distance.
