# Supplementary material for: Genome-wide association mapping revealed a diverse genetic basis of seed dormancy across subpopulations in rice (Oryza sativa L.)
Source: BMC Genet. 2016 Jan 25;17:28. doi: 10.1186/s12863-016-0340-2 (PMC4727300; doi:10.1186/s12863-016-0340-2)
Supplement: Additional file 2: — List of twenty most dormant accessions that retained their dormancy in After-ripened seeds. This table contains names of twenty most dormant accessions, sub- population, country of origin and the germination percentages in FHS and ARS. (PDF 95 kb) [file 12863_2016_340_MOESM2_ESM.pdf]

Additional file 2: List of twenty most dormant accessions that retained their dormancy in After-ripened seeds

Accession ID	Accession Name	Population	Germination Percentage		Origin
			FHS	ARS	
W104	CAROLINO 164	Aus	0.0	5.2	Chad
W142	DNJ 121	Aus	0.0	6.2	Bangladesh
W099	SL 22-620	Aus	0.0	7.2	Sierra Leone
W108	HI MUKE	Aus	2.0	7.4	Kazakhstan
W278	MOISDOL	Aus	1.7	8.1	Bangladesh
W106	DAUDZAI FIELD MIX	Aus	4.0	9.8	Pakistan
W100	SPIN MERE	Aus	0.9	12.1	Afghanistan
W040	INIAP 7	IndII	2.0	12.1	Ecuador
W180	BG300	IndII	11.6	14.1	Sri Lanka
W134	DARA	Aus	12.5	18.9	Indonesia
W279	JABOR SAIL	Aus	0.0	20.5	Bangladesh
W043	DICHROA ALEF USLKIJ	IndII	1.3	20.5	Kazakhstan
W076	TAULI	Aus	0.3	30.8	Nepal
W305	AKITAKOMACHI	Tej	15.2	31.2	Japan
C189	PUTAOHUANG	Tej	19.4	32.4	China
W168	BG90-2	IndII	8.8	37.7	Brazil
W228	OM1723	IndII	19.8	38.1	Viet Nam
W296	AUS 371	Aus	24.0	38.7	Bangladesh
W095	DJ 24	Aus	0.6	45.9	Bangladesh
C169	TAIZHONGZAILAI1	IndII	11.6	47.2	Taiwan-China
