# Supplementary material for: Genome-wide association mapping revealed a diverse genetic basis of seed dormancy across subpopulations in rice (Oryza sativa L.)
Source: BMC Genet. 2016 Jan 25;17:28. doi: 10.1186/s12863-016-0340-2 (PMC4727300; doi:10.1186/s12863-016-0340-2)
Supplement: Additional file 3: — List of most dormant accessions that lost their dormancy in After-ripened seeds. This table contains names of most dormant accessions, sub- population, country of origin and the germination percentages in FHS and ARS. (PDF 105 kb) [file 12863_2016_340_MOESM3_ESM.pdf]

Additional file 3: List of most dormant accessions that lost their dormancy in After-ripened seeds

Accession ID	Accession Name	Population	Germination Percentage		Origin
			FHS	ARS	
W286	GARIA	Aus	3.0	92.1	Bangladesh
W181	BG94-1	IndII	4.0	78.0	Sri Lanka
W242	AJAYA	IndII	5.7	97.3	India
W161	Y134	IndII	7.0	72.9	China
W313	OM 2517	IndII	9.4	90.8	Viet Nam
C011	MILYANG 23	IndII	10.2	65.3	Korea
W178	MANAWTHUKHA	IndII	12.7	94.2	Philippines
C061	YANGDAO2HAO	IndII	13.8	99.3	China
W051	RP2151-173-1-8	IndII	16.1	94.4	India
W317	CIHERANG	IndII	18.0	81.2	Indonesia
W176	MILYANG23	IndII	18.0	68.4	Korea
W291	IR 2071-625-1-252	IndII	18.6	75.1	Philippines
W292	KHOIA BORO	IndII	18.8	87.8	Bangladesh
W174	GAYABYEO	IndII	19.8	95.5	Korea
C052	GUIHUAHUANG	Tej	22.5	80.0	China
W238	UPRI91-66	IndII	24.8	98.9	India
W298	IR36	IndII	28.2	99.2	Philippines
W152	CDR22	IndII	29.7	83.6	China
W263	SADAJIRA-19-303	IndII	29.8	72.1	Unknown

C070	YOUMANGZAOGENG	Tej	32.1	81.5	China
W223	AT354	IndII	32.6	97.4	Sri Lanka
W289	IR 661-1-140-3-117	IndII	34.1	80.0	Philippines
W307	AGNO (PSBRC28))	IndII	35.6	96.0	Philippines
C056	JINDAO1HAO	Tej	36.5	77.1	China
W059	NIWAHUTAW MOCHI	Tej	37.8	81.3	Japan
C146	NIPPONBARE	Tej	38.8	88.5	Japan
W310	BRRI DHAN 29	IndII	38.9	77.5	Bangladesh
C147	MINGHUI63	IndII	41.4	83.5	China
W241	PHALGUNA	IndII	43.7	95.0	India
W067	MITAK	TrJ	45.9	94.7	Indonesia
C049	JINYOU1HAO	IndII	46.0	80.1	China
C119	NINGHUI21	TeJ	46.8	84.8	China
W153	CHENGHUI448	IndII	48.1	86.7	China
W227	OM1706	IndII	49.2	98.4	Viet Nam
W265	LA110	IndII	49.8	86.9	United states
W295	LEBONNET	IndII	50.0	83.1	United States
