# Supplementary material for: Genome-wide association mapping revealed a diverse genetic basis of seed dormancy across subpopulations in rice (Oryza sativa L.)
Source: BMC Genet. 2016 Jan 25;17:28. doi: 10.1186/s12863-016-0340-2 (PMC4727300; doi:10.1186/s12863-016-0340-2)
Supplement: Additional file 4: — Non-synonymous SNPs in Sdr4 and GA2ox3 genes in 350 accessions used in our study. This file contains two tables (a) and (b). Table (a) contains the non-synonymous SNPs in Sdr4 and GA2ox3 genes, the SNPs position within the chromosomes, the minor and major alleles for the SNPs and the Amino acid changes in relation to the nucleotide change from major to minor alleles. The tables also shows the haplotype diversity within these two genes. Table (b) shows the number of accessions in individual sub-populations possessing any of the Sdr4 and GA2ox3 haplotypes. (PDF 101 kb) [file 12863_2016_340_MOESM4_ESM.pdf]

Additional file 4 (a) : Non-synonymous SNPs in Sdr4 and GA2ox3 genes

Sdr4	*SNP ID*	*sf0723795969*	*sf0723796436*	*sf0723796212*
	Chromosome	7	7	7
	Position (bp)	23795969	23796436	23796212
	Major Allele	**A**	**G**	**C**
	Minor Allele	**G**	**C**	**T**
	Amino Acid change	T to A	A to P	A to V
	Hap1	A	G	C
	Hap2	G	C	C
	Hap3	G	C	T
GA2ox3	*SNP ID*	*sf0131794745*	*sf0131794598*	*sf0131795793*
	Chromosome	1	1	1
	Position (bp)	31794745	31794598	31795793
	Major Allele	**C**	**G**	**C**
	Minor Allele	**G**	**A**	**T**
	Amino Acid change	L to V	V to I	A to V
	Hap1	C	G	C
	Hap2	C	G	T
	Hap3	G	G	C

Additional file 4: **(a)** Non-synonymous SNPs in Sdr4 and GA2ox3 genes in 350 accessions used in our study. Amino acid changes are indicated in relation to the nucleotide change from major to minor alleles. The SNPs in each gene resulted into three haplotypes (Hap1-3).

Additional file 4 (b): Number of Accessions in sub-populations and their individual haplotypes in Sdr4 and GA2ox3

Gene	*Haplotype*	*IndI*	*IndII*	*Aus*	*Tej*	*Trj*
		Number of accessions	Number of accessions	Number of accessions	Number of accessions	Number of accessions
Sdr4	*Hap1*	57	20	21	45	37
	Hap2	3	47	9		
	Hap3	1	14			
GA2ox3	*Hap1*	34	82	20	11	
	Hap2	24		10		
	Hap3	1			45	26

Additional file 4: **(b)** The number of accessions in sub-populations of indica, Aus and japonica rice possesing any of the three haplotypes of *Sdr4* and *GA2ox3*. IndI, indica I; IndII, indica II; Tej, temperate japonica; Trj, tropical japonica.
