# Supplementary material for: Genome-wide association mapping revealed a diverse genetic basis of seed dormancy across subpopulations in rice (Oryza sativa L.)
Source: BMC Genet. 2016 Jan 25;17:28. doi: 10.1186/s12863-016-0340-2 (PMC4727300; doi:10.1186/s12863-016-0340-2)
Supplement: Additional file 5: — Estimated effective number of SNPs and significant Thresholds in populations: This table shows the effective number of independent SNPs (Me) after a modified Bonferroni correction calculated using informative SNPs (M) in Whole population, Aus, indica and japonica populations. (PDF 85 kb) [file 12863_2016_340_MOESM5_ESM.pdf]

**Additional file 1 Estimated effective number of SNPs and significant Thresholds in populations**

| *Population* | *Observed SNPs* | *Effective Number (Me)* | *Effective ratio* | *Suggestive P-Value* | *Significant P-value* |
|---|---|---|---|---|---|
| *All* | 3916415 | 757577.8 | 0.19 | 1.32E-06 | 6.60E-08 |
| *Aus* | 1925362 | 235880.1 | 0.12 | 4.24E-06 | 2.12E-07 |
| *indica* | 2767159 | 571842.8 | 0.21 | 1.75E-06 | 8.74E-08 |
| *japonica* | 1857845 | 245347.5 | 0.13 | 4.08E-06 | 2.04E-07 |
