# Supplementary material for: Genome-wide association mapping revealed a diverse genetic basis of seed dormancy across subpopulations in rice (Oryza sativa L.)
Source: BMC Genet. 2016 Jan 25;17:28. doi: 10.1186/s12863-016-0340-2 (PMC4727300; doi:10.1186/s12863-016-0340-2)
Supplement: Additional file 7: — The Genome-wide association mapping results for Germination Percentage (GP) of the after-ripened seeds (ARS) in whole, Aus, indica and japonica populations. The figure shows neighbor-joining tree, histogram of the phenotypes (GP), quantile-quantile plot of the expected null distribution and the observed P-value and the Manhattan plots of GP of after-ripened seeds in populations using LMM and LR methods. (PDF 823 kb) [file 12863_2016_340_MOESM7_ESM.pdf]

Trait: GP **Pop:** *All*

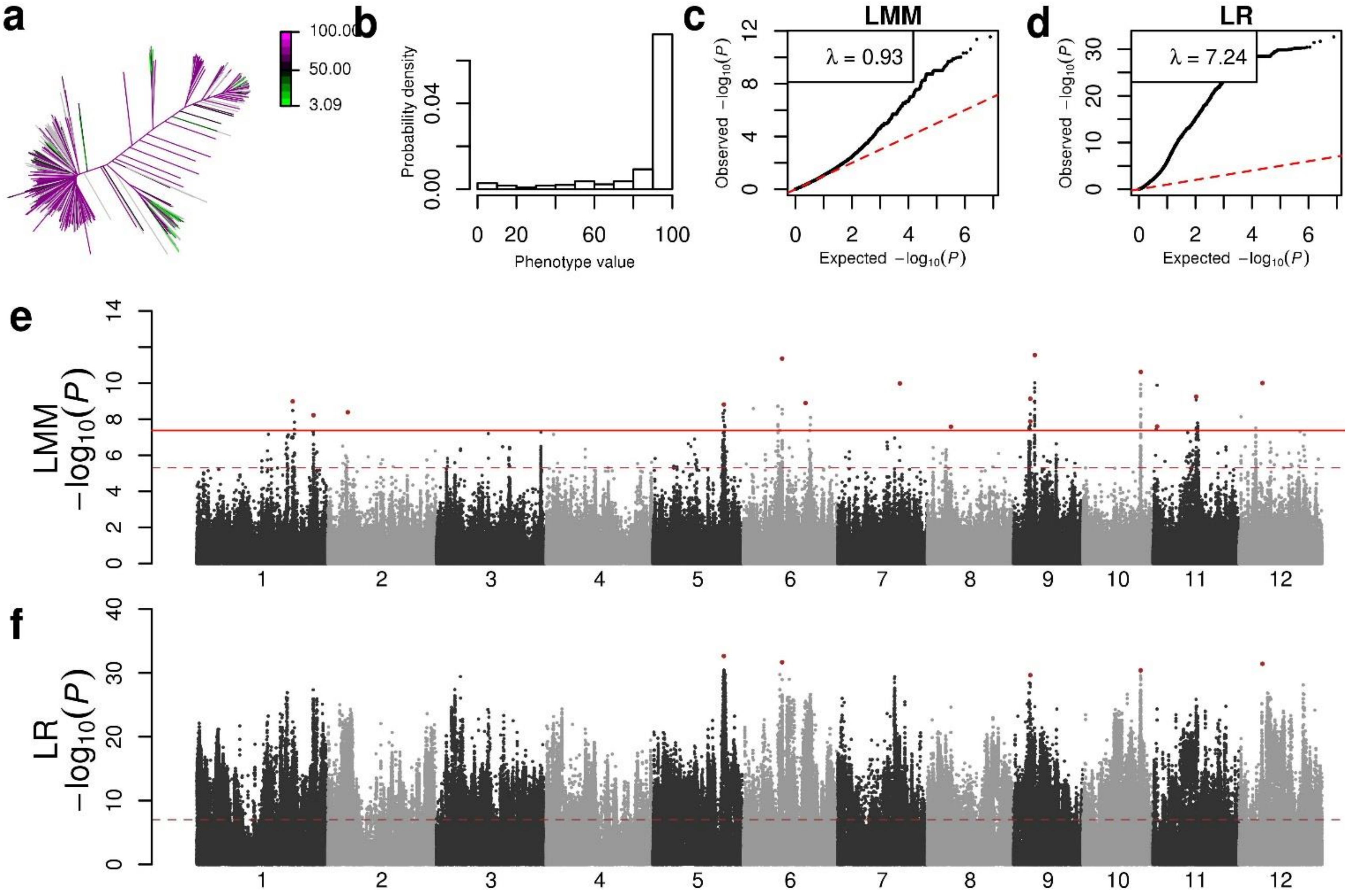

Trait: GP **Pop:** *Aus*

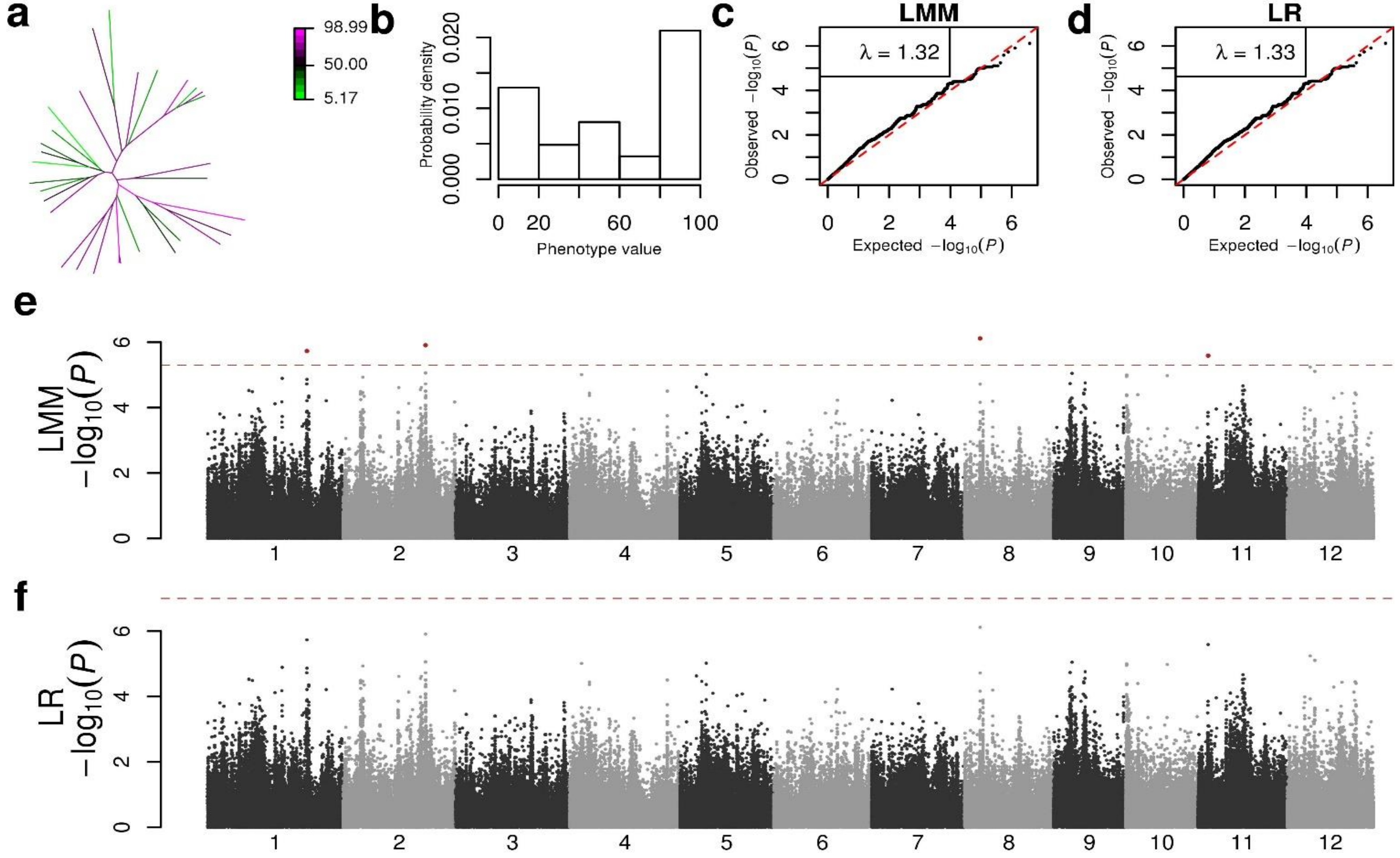

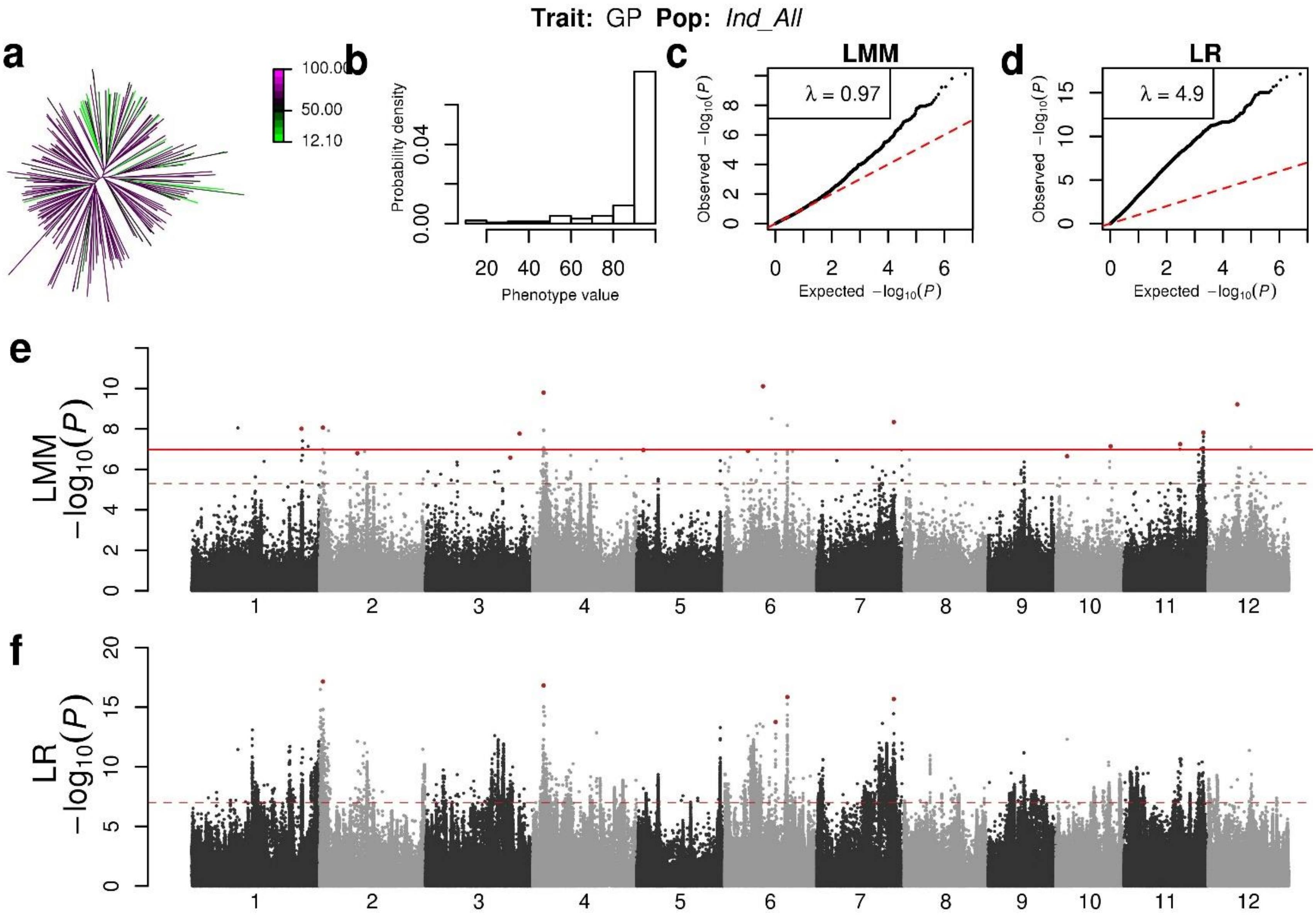

Trait: GP Pop: Ind_All
a
100.00
50.00
12.10
b
Probability density
0.00
0.04
20 40 60 80
Phenotype value
c
LMM
λ = 0.97
Observed −log10(P)
Expected −log10(P)
d
LR
λ = 4.9
Observed −log10(P)
Expected −log10(P)
e
LMM
−log10(P)
1 2 3 4 5 6 7 8 9 10 11 12
f
LR
−log10(P)
1 2 3 4 5 6 7 8 9 10 11 12

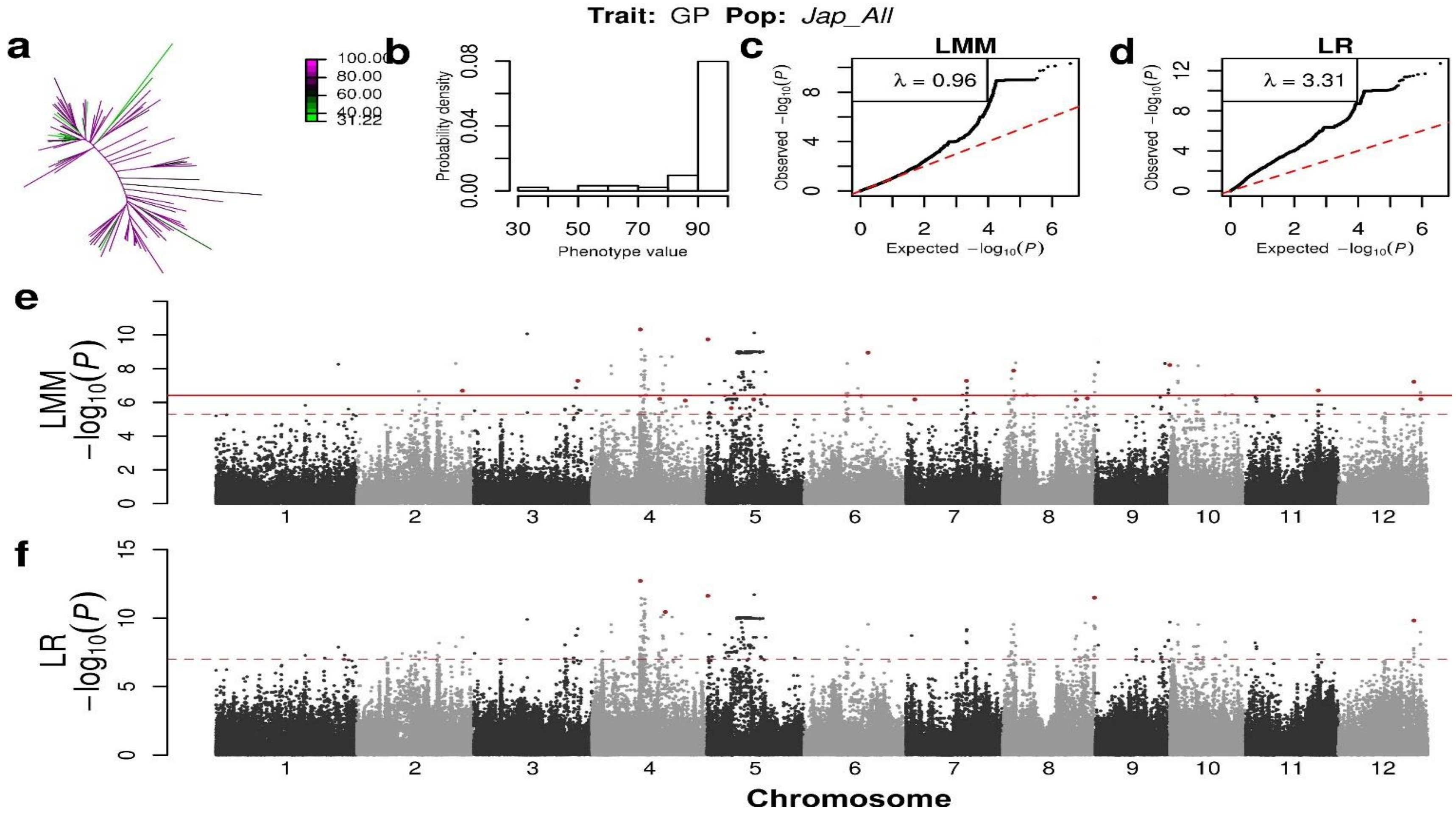

Additional file 7: The GWAS analysis results and quantile-quantile (Q-Q) plots of p-values for germination percentage (GP) of after-ripened seeds (ARS) in whole, Aus, indica and japonica populations in our study.

a) Neighbor- joining tree of rice varieties in a corresponding population. **(b)** A histogram of the phenotype in the population **(c-d)** Quantile- Quantile plot of the expected null distribution and the observed P-value using the Linear mixed model **(c)** and linear regression model **(d). (e-f)** Genome-wide P-values for the mixed model **(e)** and the simple linear regression model.
